# Hierarchical Vision Navigation System for Quadruped Robots with Foothold Adaptation Learning

**DOI:** 10.3390/s23115194

**Published:** 2023-05-30

**Authors:** Junli Ren, Yingru Dai, Bowen Liu, Pengwei Xie, Guijin Wang

**Affiliations:** 1 Department of Electronic Engineering, Tsinghua University, Beijing 100084, China; rjl21@mails.tsinghua.edu.cn (J.R.);; 2 Shanghai Artificial Intelligence Laboratory, Shanghai 200232, China

**Keywords:** vision system, robot navigation, motion planning

## Abstract

Legged robots can travel through complex scenes via dynamic foothold adaptation. However, it remains a challenging task to efficiently utilize the dynamics of robots in cluttered environments and to achieve efficient navigation. We present a novel hierarchical vision navigation system combining foothold adaptation policy with locomotion control of the quadruped robots. The high-level policy trains an end-to-end navigation policy, generating an optimal path to approach the target with obstacle avoidance. Meanwhile, the low-level policy trains the foothold adaptation network through auto-annotated supervised learning to adjust the locomotion controller and to provide more feasible foot placement. Extensive experiments in both simulation and the real world show that the system achieves efficient navigation against challenges in dynamic and cluttered environments without prior information.

## 1. Introduction

Recently, legged robots have demonstrated agile locomotion advantages over wheeled robots, especially when walking in various challenging scenes with obstacles such as thresholds, steps, furniture, and pedestrians. Quadruped robots can perform various motions [1] and undertake various dangerous and dirty tasks such as disaster relief and mine exploration [2]. Thus safe and efficient navigation is promising in the study of quadruped robots. Navigation on quadruped robots aims at reaching the target with few collisions [3]. The task is usually decoupled into two parts: A navigation policy planning the desired path that provides the Center of Mass (COM) velocity, and a low-level locomotion controller generating the appropriate joint torque and optimal leg swing trajectory for each step.

Traditionally, navigation policies construct maps through comprehensive visual perception [4,5,6], causing the navigation performance to fluctuate with different vision systems. Moreover, errors will be accumulated when constructing maps without prior knowledge in unknown environments [7]. Therefore, end-to-end learning policies that directly use egocentric vision have been implemented in robot navigation [8], minimizing the intermediate steps between environment perception and locomotion control. Once the optimal path is generated, practical navigation tasks require the robot to follow the planned trajectory. However, traditional navigation policies replace the accurate robot model with an idealized cylindrical agent (ICA) when planning COM motion [8]. Since these policies do not take the dynamic characteristics of the robot into account, they fail to control a specific robot smoothly to follow the planned trajectory in cluttered scenes [9]. Thus, successful navigation requires the combination of the low-level locomotion controller.

**Figure 1:**
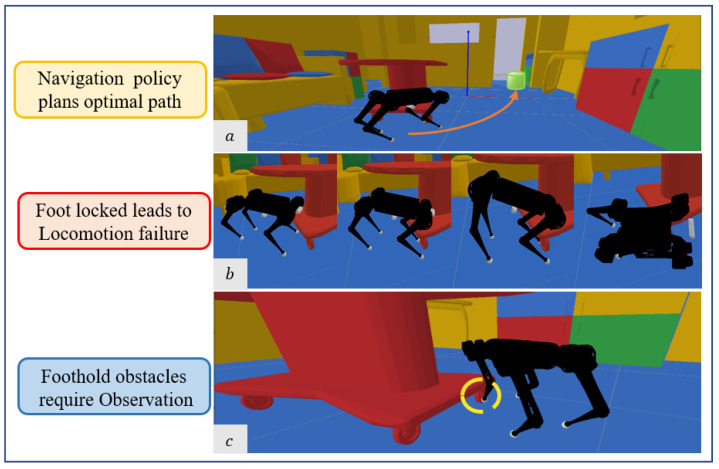
Challenges for quadruped robot navigation in cluttered environments: (**a**) Traditional front observation captures obstacles at the front and plans avoidance. (**b**) Since the forward-facing camera has no perception of obstacles around the foothold, the locomotion control may fail when the leg becomes stuck. (**c**) The obstacles can be spotted with foothold observation.

A proliferation of works has developed locomotion controllers for robot navigation. Traditional legged controllers [10,11,12] based on proprioception optimization lack knowledge of the environment. Thus, the robot is unable to traverse dynamic scenes. Therefore, recent works have introduced external visual feedback into the locomotion controller and have made continuous foothold adaptation based on local observation [13,14,15,16]. As heuristic methods require prior information on the terrain, learning-based adaptation policies with onboard sensors have shown greater potential. However, existing perception methods of mobile robots seldom consider dynamic foothold adaptation while navigating in cluttered environments. As shown in Figure 1, the robot gets stuck at challenging obstacles, while the vision sensors in the moving direction are not aware. Therefore, designing a vision acquisition system to tackle difficult locomotion tasks such as navigation in cluttered scenes is a great challenge.

In this paper, we propose a novel hierarchical vision system to navigate a quadruped robot in indoor scenarios with challenging obstacles. More specifically, the system is composed of a Front Camera receiving forward-facing depth information and a Foothold Camera receiving gray-scale foothold observation. Based on the Front Camera, the high-level navigation policy plans an optimal path toward the goal and generates COM velocity commands. Based on the Foothold Camera, the low-level policy trains the foothold adaptation policy through auto-annotated supervised learning to inform the locomotion controller of the appropriate foothold. Furthermore, based on the small-scale quadruped robot (MIT mini-cheetah), we implement the practical hierarchical vision system on a hardware platform, including visual perception and onboard computing devices. Extensive experiments in both simulation and the real-world show that the system achieves efficient navigation against the challenges in dynamic and cluttered environments without prior information. The videos can be found in Supplementary Materials Section.

The main contributions can be summarized as follows:

(1) The proposed hierarchical vision system combines high-level navigation policy with vision-based locomotion control, resulting in a data-efficient and concise embodied system with increased practical applicability for legged robots.
(2) We design an auto-annotated supervised training approach for a foothold adaptation policy that optimizes a visual-based controller without complex reward designs or extensive training; the policy establishes a direct correlation between foot observation and successful obstacle avoidance.

## 2. Related Work

We develop the original hierarchical vision navigation system based on recent progress in quadruped navigation and locomotion control. This section introduces the related literature in this area.

### 2.1. Vision Sensors for Robot Navigation

The ability to perceive visual cues is essential for quadruped robots to accomplish optimal obstacle avoidance and successful navigation. Conventional navigation algorithms rely on visual sensors such as laser radars and front cameras to construct a map of the environment, and then employ path planning algorithms such as A* and DWA based on the map [17,18,19,20]. However, such vision systems are often characterized by significant blind spots, particularly for robots with intricate mechanical structures. Although other vision systems such as spinning radar [21] or multi-camera arrays surrounding the robot [22] have been proposed, they are either insufficient for quadruped robots, as they fail to capture the critical observation of footholds necessary for safe locomotion, or seem redundant when considering an efficient visual system.

To make more efficient use of the external visual information necessary for successful navigation in common indoor environments, we propose a novel hierarchical vision system that provides simultaneous forward and local foothold views.

### 2.2. Quadruped Robots Navigation Policy

Map-based navigation policy finds the optimal path through heuristic search, where an efficient SLAM algorithm is usually required when constructing the map for path planning [4,23,24,25,26]. Other approaches such as Deep Reinforcement Learning (DRL) directly generate velocity commands through state representation and combine the state with pixels from onboard visual sensors [27,28,29].

The generic navigation methods assume the agents to follow the planned path perfectly [30,31]. However, various robots act differently upon receiving the same COM velocity command [32]. Therefore, specific adaptation to the COM velocity command is required for quadruped robot navigation. Some works decouple the navigation policy and the low-level locomotion controller [33], while combined frameworks have also been proposed, such as introducing specific embeddings [8] and coupling vision state with proprioception [34].

Different from previous works, we combine the end-to-end navigation policy with an active foothold adaptation mechanism. The combined navigation framework receives external perception information from the proposed hierarchical vision system, improving the performance of the quadruped robot to follow the navigation COM velocity commands.

### 2.3. Quadruped Robots Foothold Adaptation

In earlier works, quadruped robots have achieved stable locomotion capabilities through the exploration of proprioceptive states such as Model Predictive Control (MPC) and Whole Body Control (WBC) [35]. These methods calculate foothold by optimizing the ground contact force. Vision input has also been leveraged into the locomotion controller for feasible foothold selection, where various visual caption systems have been proposed. Traditional approaches mount onboard radar and stereo cameras to build a terrain heightmap locally and to select a traversable foothold through potential search [36,37].

Recently, reinforcement learning (RL) has been widely developed in leading-edge works to build learning-based controllers [38,39,40] or to make foothold adaptations based on a model-based controller [14,41]. However, RL has limitations that remain unsolved, such as the difficult and time-consuming process of designing appropriate reward functions, extensive training requirements, and high computational costs. Furthermore, transferring RL learned policies to real robots is a new challenge, requiring additional modules such as knowledge distillation [42,43] or imitation learning [44]. Unlike prior works that use RL to predict feasible foothold adaptations, our adaptation policy is developed by learning a network trained through auto-annotated supervised learning, which can be optimized using automatically labeled lightweight data. Additionally, our adaptation policy directly takes egocentric images as input, in contrast to state-of-the-art approaches that build local heightmaps [13,14,34] as an intermediate step.

## 3. Hierarchical Vision Navigation System

**Figure 2:**
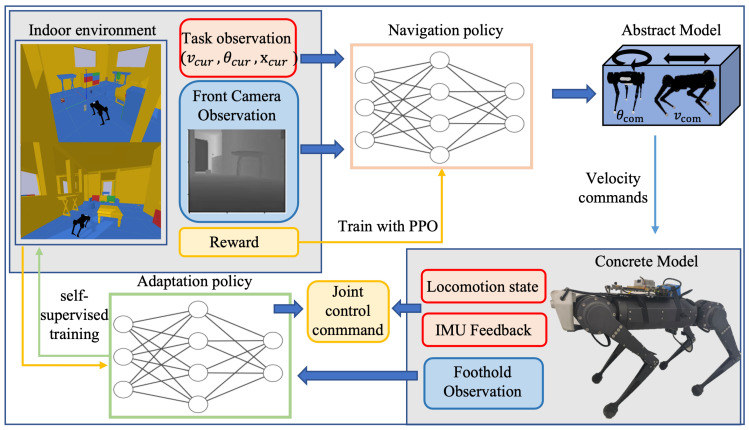
Overview of the proposed navigation framework. The navigation policy is trained with the abstract model of the robot in the cluttered indoor environment. The navigation policy generates velocity commands to the robot concrete model to approach the target point. Based on the Foothold observation, we combine the low-level locomotion controller with a foothold adaptation policy to control the robot’s following the optimized velocity commands smoothly.

Herein, we introduce our hierarchical vision navigation system, which synergistically combines active foothold adaptation and end-to-end navigation policy for optimal performance in cluttered indoor scenes. For task observation, the robot is only provided with the location of the target point and the perception of its locomotion state from an onboard ego-motion estimator. For environment observation, the Front Camera and the Foothold Camera offer egocentric visual feedback from two perspectives. The overall framework of the novel-designed navigation system is illustrated in Figure 2.

The high-level policy receives depth information from the front and task observation. A convolutional encoder is used to extract the visual features $l_{\mathrm{depth}}$, which are merged with $l_{\mathrm{task}}$ and passed into a multilayer perceptron (MLP). The MLP then generates COM velocity from $S_{t}$. The navigation policy is learned using reinforcement learning. Rewards are obtained through each step of policy optimization, among which the potential reward requires information from the global map. The velocity commands control an abstract model of the robot to interact with the cluttered indoor scene to update the navigation policy.

On the level of foothold adaptation, different from previous research on legged control with terrain information [14,37], our proposed system leverages foothold observations using auto-annotated supervised learning to train a classification policy. This policy enables the robot to identify safe footholds, improving its ability to follow the centroid velocity output of the high-level navigation policy. Besides foothold observation, the enhanced locomotion controller receives the robot locomotion states (joint position and joint velocity) and the IMU sensor feedback to calculate the desired joint position. The locomotion controller and adaptation policy interact with the robot concrete model in both the simulation and the real world.

Our proposed system enables efficient navigation through indoor environments by selecting appropriate footholds to avoid obstacles, rather than requiring detours. To evaluate its performance, we use point-goal navigation, which involves navigating towards a specific goal location while avoiding obstacles [45]. We incorporate a variety of small indoor obstacles into the scenes using existing navigation datasets [46] to simulate challenging realistic navigation conditions.

### 3.1. Navigation Policy

Our high-level navigation policy implements the approach in [46] on the quadruped robot. To optimize the navigation policy for the quadruped robot, we employ the use of an abstract model to interact with the environment. Rather than relying on the idealized cylindrical agent (ICA), this approach allows for more efficient training of the robot’s navigation policy. The end-to-end policy is trained through reinforcement learning to receive egocentric visual feedback. The Front Camera captures depth frames at a resolution of 128×128 to extract obstacles observation. The camera has a field angle (FOV) of 60 degrees to detect distance clipped at [0.5 m, 5 m]. We build up the action space to keep the robot moving in a consistent direction with the Front Camera. The discrete action space includes linear velocities $v_{\mathrm{com}}$ (forward or backward) in the range of [−0.3 m/s, 0.3 m/s], and angular velocities $\theta_{\mathrm{com}}$ (clockwise and counterclockwise) in the range of [$-\frac{\pi }{6}$ rad/s, $\frac{\pi }{6}$ rad/s]. The robot has to turn to the new heading and then move forward when moving laterally.

For task observation $\left(v_{\mathrm{cur}},\theta_{\mathrm{cur}},x_{\mathrm{loc}}\right)$, we combine the robot base velocity with the relative target location, where $x_{\mathrm{loc}}$ is the relative displacements between the robot and the target point. All of the task observation is calculated through the state estimator from the original IMU output obtained directly from the onboard inertial measurement unit (IMU).

The high-level policy is trained through Deep Reinforcement Learning (DRL). We use PPO [47] as the training approach of the COM velocity predictor. The action is predicted as a 2-dimensional vector $\left(v_{\mathrm{com}},\theta_{\mathrm{com}}\right)$. We transfer the standard reinforcement learning framework provided by RLlib [48] to implement the DRL training.

Without any artificial design, we set dense rewards to guide the robot in planning the optimal path from the observation space. The dense rewards consist of two parts: the potential reward, which measures the distance from the current location to the target, and the collision reward, which evaluates the safety of the navigation policy. A success reward is also obtained after each episode if the robot reaches the goal:

(1) $$r_{t}=r_{\mathrm{potential}}+r_{\mathrm{success}}+r_{\mathrm{collision}},$$

(2) $$r_{\mathrm{potential}}=\omega_{0}\times \left(d_{t}-d_{t-1}\right),$$

(3) $$r_{\mathrm{collision}}=\omega_{1}\times n_{\mathrm{collision}},$$

where $r_{\mathrm{success}}$ is the success reward obtained when reaching the target point; $r_{\mathrm{collision}}$ is the collision reward used as the penalty for the robot after a collision with the obstacles. $r_{\mathrm{potential}}$ is the potential reward, examining the shortest path length of the robot to reach the target point from the current location. $d_{t}$ is the distance from the current location of the robot to the target point at time *t*.

$\omega_{0}$ and $\omega_{1}$ regularize each of the reward terms. Given the premise of $\omega_{0}<0$ and $d_{t}<d_{t-1}$, we see $r_{\mathrm{potential}}>0$, which indicates that the robot moves closer to the target point. Meanwhile, to obtain the collision reward in the training process, we replace the idealized cylindrical agent (ICA) with a rectangle model of (0.485 m, 0.275 m, 0.3 m), which is the size of the bounding box of the quadruped robot.

### 3.2. Controller with Foothold Adaptation

The implemented navigation policy enables the quadruped to find an optimal path to the target. Since the Front Camera is installed on the quadruped robot in a fixed orientation, the robot’s locomotion could be impeded when the foot is locked by the tiny obstacles around. To address this issue, our proposed system incorporates a Foothold Camera that captures the visual data of small obstacles in the blind areas of the Front Camera. The COM velocity generated from the high-level navigation policy is converted into the desired foothold, where our foothold adaptation module provides dynamical modification.

We mount the foothold Camera with a tilt angle to allow for effective observation of footholds during the navigation task. The policy was trained through auto-annotated supervised learning techniques that leverage the robot’s interaction with the cluttered indoor scenes. Specifically, the adaptation action and their effects were used to generate training data without the need for manual annotation or external supervision. With the adapted foothold, the robot then generates desired leg swing trajectory by considering the foothold as the end point of the trajectory and calculates the desired joint torque.

## 4. Foothold Adaptation Learning

**Figure 3:**
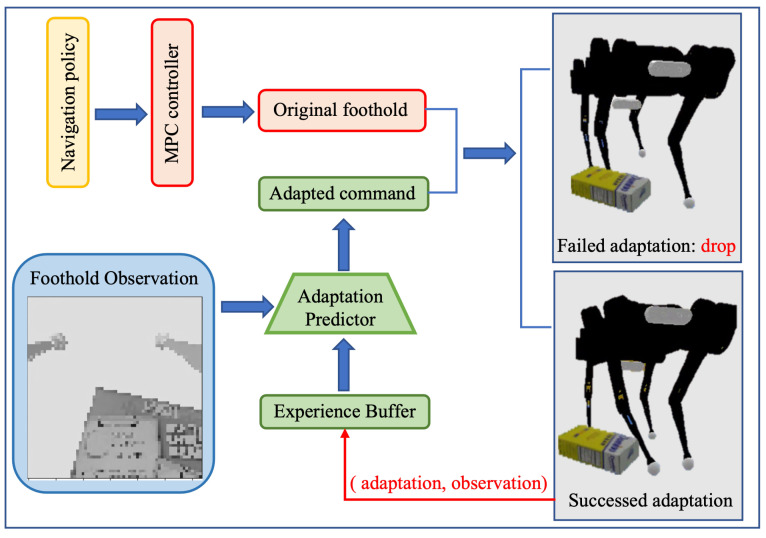
The foothold adaptation policy records successful adaptation actions and their corresponding observations into an experience buffer, which is then utilized to generate labels for the adaptation predictor.

In this section, we introduce the proposed foothold adaptation policy in detail. Our method utilizes exteroceptive information in the locomotion controller in a different way from VisionMPC [36]. Since VisionMPC requires the heightmap construction, our adaptation policy combines the locomotion controller with an auto-annotated supervised advisor that generates foothold adaptation commands directly. As shown in Figure 3, the proposed foothold adaptation policy adapts the original footholds computed by the MPC controller using predictions from the adaptation policy to generate desired footholds. The locomotion controller then utilizes these desired footholds to calculate the joint torque of each leg. Once a successful control step is finished (we define a step without collision as a successful step), the adaptation policy records the label and is updated automatically, while the failed adaptation actions are dropped. The enhanced controller enables the robot to traverse cluttered and dynamic environments without any prior information.

The quadruped robot applies MPC and WBC within the low-level locomotion controller [35]. Under the assumption that the legs contact the ground with a fixed period, these controllers optimize the ground contact force and calculate the joint torques through inverse kinematics. To overcome the challenge of unexpected obstacles in cluttered environments that can lead to a deviation between the assumed and actual situations, causing locomotion failure, the proposed foothold adaptation policy utilizes observations around foot placement to minimize leg contact with obstacles.

### 4.1. Observation System and Adaptation Action

**Figure 4:**
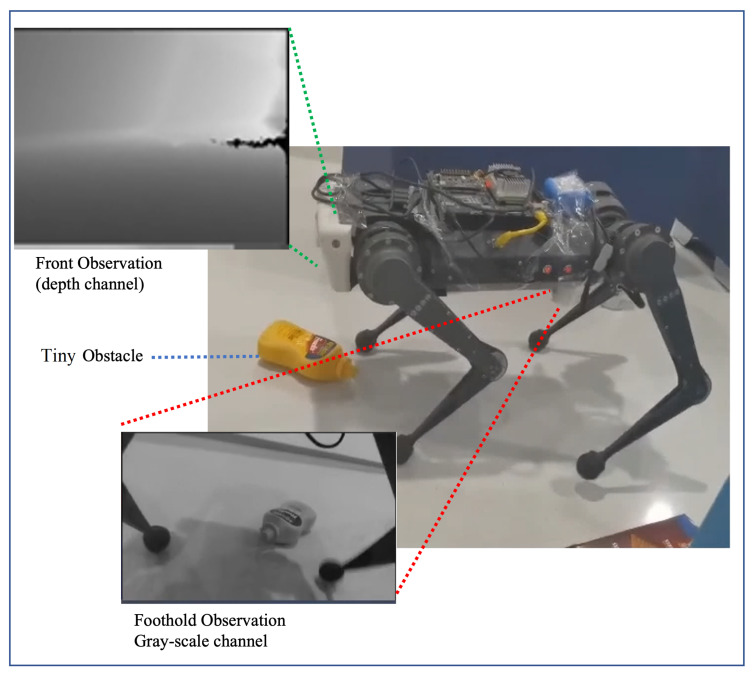
The designed hierarchical vision system captures foothold and front observation simultaneously. Challenging obstacles are in sight of the Foothold Camera.

**Figure 5:**
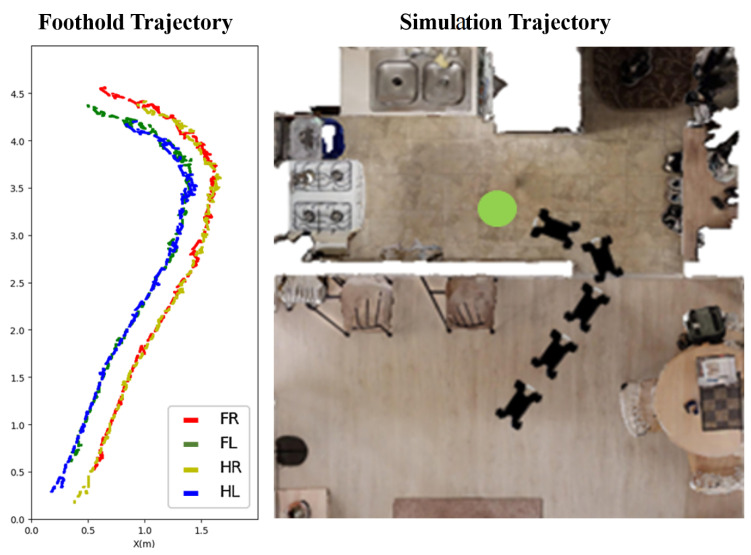
(**left**): Foothold trajectories of the four legs. The trajectory of the hind foot approximately follows the front foot. (**right**): Corresponding trajectory for the quadruped robot’s navigation in the simulation, in indoor scenes.

Since the Front Camera lacks observation of the foot placement, the proposed hierarchical vision system introduces the Foothold Camera to capture information around the footholds, which is shown in Figure 4. This is different from previous work, which implements two cameras to observe both the hind and front foothold [41], or constructs a terrain map with rotating sensors [16]. We have observed that the safe foothold planned by the front leg remains safe for the rear leg (see Figure 5) in a typical navigation scenario. Thus, we propose adjusting the hind foothold by imitating the action of the front, based on the observed similarities in safe foothold planning between the two legs. The low control frequency of the foothold adaptation policy justifies this approximation. Based on the above discussion, we have streamlined our system by mounting a single camera to capture the observation of the front foothold.

While map-based foothold adaptation methods rely on constructing heightmaps and searching neighboring grid locations to find feasible footholds in cluttered scenes [36,49], our proposed policy employs a different approach. Instead, we train a foothold adaptation policy without constructing a heightmap, and we design adaptation actions for the four legs as relative displacements from the foothold generated using MPC (Original Foothold). For each leg, the displacement $A_{t}$ is a vector with different orientations. The lengths of these vectors are limited to 0.1 m in the XY plane and 0.03 m in the z-component.

### 4.2. Policy Learning

We construct the adaptation policy with a classification network. To more effectively obtain successful foothold adaptation, we alternate between data collection and network updates. More specifically, auto-annotated supervised learning is conducted in an ϵ-greedy manner. The robot applies random footholds under the probability of ϵ and applies current policy output under the probability of 1−ϵ. A new data sample is added if the current step has no collision with the obstacles, and the network is updated once a certain amount of samples have been added (Algorithm 1). In addition, we constantly modify the cluttered environment with different challenging obstacles during simulation training, enabling the robot to adapt to different types of complex scenarios. It should be noted that during data collection, the robot was allowed to freely select $A_{t}$ in the area of ([−0.1, 0.1], [−0.1, 0.1], [0, 0.3]). However, to fit the data into the classification network, we discretized the action space uniformly into eight label positions for each of the front foot. We used the nearest neighbor principle to find the label position that was closest to the acted $A_{t}$. This approach ensured that the adaptation labels were recorded accurately and efficiently.
**Algorithm 1** Adaptation Policy Routine**Require:** adaptation action library A, greedy factor ϵ1:Initialize: adaptation policy π, experience data buffer B   *Learning Process*2:**for** epoch i=0,1,...,N **do**3:   **while** not Success and not Done **do**4:     collect gray-scale image $I_{t}$5:     **if** (random([0,1])<ϵ) **then**6:        random choose $A_{t}$ from the action library A7:     **else**8:        $A_{t}=\pi \left(I_{t}\right)$9:     **end if**10:     **if** (no collision) **then**11:        // record successful adaptations12:        append current data $\left(A_{t},I_{t}\right)$ into B13:     **end if**14:   **end while**15:   // UPDATE POLICY16:   update π with updated experience data buffer B17:**end for**18:**return** π


### 4.3. Combination with Locomotion Controller

We integrate the learned policy with the MPC locomotion controller [22] of the quadruped robot, converting the adaptation commands into the desired foothold location. The combined low-level controller also achieves stable results in the real world.

With the desired COM velocity $\left(v_{\mathrm{com}},\theta_{\mathrm{com}}\right)$, the locomotion controller generates a set of coordinates of the foothold without exteroceptive information, which can maintain a stable attitude and follow the desired velocity on flat terrain. However, in cluttered indoor scenes, the original footholds cause the robot to collide with the obstacles, and the desired velocity cannot be followed. We add the adaptation policy outputs to the static coordinates to calculate the adjusted foothold:

(4) $$x_{f}=x_{\mathrm{pre}}+v\Delta t+R_{z}\left(\psi \right)\left(A_{t}+d_{l}\right),$$

where $x_{f}$ is the desired foothold location with respect to the world frame. v is the current velocity vector calculated from $\left(v_{\mathrm{com}},\theta_{\mathrm{com}}\right)$ and $x_{\mathrm{pre}}+v\Delta t$ calculates the updated COM location. $d_{l}$ is the original foothold location with respect to the robot COM location and $A_{t}$ is the adaptation action referring to the displacement vector. $R_{z}\left(\psi \right)$ is a rotation matrix translating angular velocity $\theta_{\mathrm{com}}$ in the global frame.

## 5. Experiment

Based on a small-scale quadruped robot, we evaluate the effectiveness of the designed vision navigation system in both simulation and the real world. Experiments show that the hierarchical vision system outperforms baselines [36,41,46] from the literature in completing target point navigation tasks with challenging obstacles.

### 5.1. Experimental Setup

#### 5.1.1. Simulation Setup

We implement a mini-cheetah URDF (Unified Robot Description Format) model with the proposed hierarchical vision system into the iGibson [46] environment in the Pybullet [50] physics engine. The locomotion controller and physics engine operate at a synchronized frequency of 500 Hz. The high-level navigation policy has an average inference time of 12 ms, while the foothold adaptation policy has an average inference time of 8 ms. These two networks run in parallel and jointly provide output to the locomotion controller at a frequency of 13 Hz. The locations of the robot and the target point are randomly generated for each episode, and the initial distance is limited to [3 m, 7 m]. Apart from the common furniture obstacles included in the dataset, we set five YCB objects [51] on the floor. As long as the robot moves 1 m, the YCB objects and the target point will randomly update their location within 1 m from the robot. In dynamic scenes, the YCB objects and target move at a maximum speed of 0.3 m/s.

#### 5.1.2. Physical Hardware

**Figure 6:**
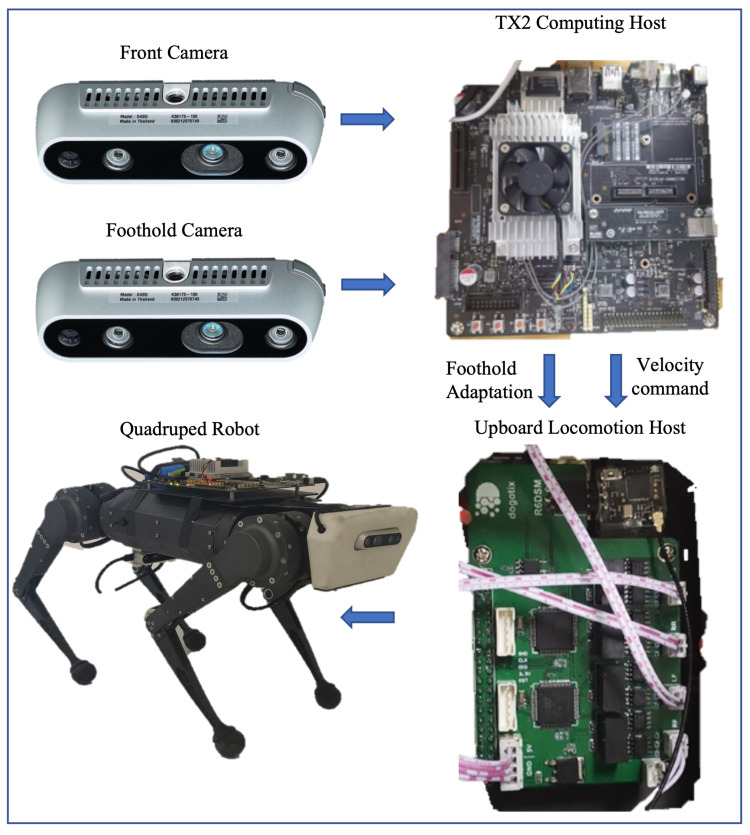
Onboard sensing and computation platform for the real-world implementation of the designed hierarchical vision system. Two D435i cameras are used as Front Camera and Foothold Camera. An NVIDIA Jetson TX2 powered by onboard supply is mounted for inference of the neural network (computing host). An Upboard computer is used for locomotion control of the robot.

The quadruped robot platform is a fork of the MIT mini-cheetah, which contains three joint motors for each of the four legs. An IMU sensor at the center of the robot body provides the original proprioception state. The proprioception state is used to calculate the task observation for high-level navigation policy, and the feedback for low-level locomotion control. We deploy our hierarchical vision system on the robot with two Intel Realsense D435i cameras which have RGB and depth channels, as shown in Figure 6. An NVIDIA Jetson TX2 powered by an onboard supply is mounted on the robot as the computing host, which is used for image processing and the inference of the neural network. The Upboard computer is mounted as the locomotion host receiving the proprioception state and sending commands to the motors. The TX2 performs inferences with an average time of 47 ms and 36 ms for the two networks, respectively, and sends the results to Upboard at 8 Hz through LCM (Lightweight Communications and Marshalling). The proposed policies demonstrate real-time performance on the embodied system without significant delay.

#### 5.1.3. Baseline and Metrics

To evaluate the navigation performance with the hierarchical vision system, we compare our method with three baselines:

(1) End-to-end navigation policy (Base-Nav) [46]: We deploy the navigation policy directly on the quadruped robot.
(2) Heuristic foot placement policy (Heuristic) [36]: Among recent foothold adaptation methods that use potential search [16,36,37], we choose to compare our method with [36] as it shares the same motion controllers with our approach. To implement this baseline, we construct a heightmap using the initial obstacle positions, and the robot autonomously chooses foothold positions with a height below 0.03 m.
(3) RL learned foothold adaptation policy (RL) [41]: We train a foothold adaptation policy using reinforcement learning as a baseline for comparison. Because we use the egocentric image input as well, we mainly replicate the RL method of [41] instead of [14] for comparison. In addition to the foothold observation, we incorporated the robot’s joint states, base velocity, and body height as inputs. For reward shaping, we incentivized speed following and penalized collisions with obstacles.

Both the Heuristic and RL policies are combined with the high-level navigation policy for evaluation.

Our metrics include:

(1) Success weighted by Path Length (SPL) [45], which evaluates the efficiency of the navigation (static target only).
(2) Success Rate (Success), which indicates the performance of the proposed system to complete the task. We consider a successful navigation to have occurred when the robot reaches the target location within twice the agent’s width (2×0.4875) [45].
(3) Crash Rate (Crashes), which is used to represent the probability that the robot will collide with the environment and cause the task to fail.
(4) Average Collisions (Collisions), which is the average number of collision points between robots and obstacles per step. The Average Collision evaluates the obstacle avoidance capability of the navigation system.

### 5.2. Implementation Details

#### 5.2.1. Network Structure

The navigation policy of our proposed system processes depth observation using a 2-D CNN to obtain $l_{\mathrm{depth}}$. The layer configurations include input channel number, output channel number, kernel size, and stride of (1, 32, 8, 4), (32, 64, 4, 2), and (64, 64, 3, 1), with a zero-padding of (2, 2, 2, 2) and (1, 1, 1, 1) for the first two layers. Task observation is processed using a 2-layer MLP with (3, 256) and (256, 256), resulting in $l_{\mathrm{task}}$. The flattened $l_{\mathrm{depth}}$ is merged with $l_{\mathrm{task}}$ and passed through a final MLP with three fully connected layers (128, 128); all the networks mentioned in the navigation policy are optimized using reinforcement learning. For the foothold adaptation policy, we adopt the ResNet34 structure [52] directly.

#### 5.2.2. Training Details

The navigation policy is trained using PPO in eight parallel processes on RLib [48] for 1200 episodes. Then, the foothold adaptation module is trained using supervised learning with on-policy data using the greedy strategy. In the training of the adaptation policy, we first collect a dataset of size 1000 without updating the network; this allows us to explore the distribution of labels in the collected data. Then, we select four successful adaptations $\left(A_{\mathrm{tright}},A_{\mathrm{tleft}}\right)$ with the highest frequency of occurrence as the final category labels. Next, whenever 100 successful adaptations are observed, the foothold adaptation policy is updated for 100 epochs. The training process continues until a total of 2000 data samples are collected, which takes approximately four hours. All the policies mentioned are trained on a desktop computer with 12 Intel Xeon processors clocked at 2.4 GHz and an NVIDIA TITAN X graphics card. Other hyperparameters are listed in Table 1.

### 5.3. Simulation Experiments

We present experiments with dynamic and static scenes separately. The dynamic scenes include dynamic targets and dynamic obstacles. For all of the evaluated approaches, the robot conducts 500 PointNav tasks in five indoor scenes according to the settings above. All the test scenes are different from the scenes used for training.

The results of the static targets are shown in Table 2. With both the static and dynamic obstacles, the proposed navigation system significantly outperforms Base-Nav, indicating that correct foothold adaptation can significantly improve the robot’s performance to avoid obstacles. Since Base-Nav is trained for sampling an optimal path with an idealized cylinder agent (ICA), the quadruped robot becomes stuck as long as it encounters obstacles beyond the view of the Front Camera. The results also demonstrate the difficulty of navigating a robot without consideration of the robot dynamics.

Compared with the heuristic foot placement approach (Heuristic), our approach is able to achieve a higher SPL and Success Rate along with lower Average Collisions. In dynamic scenes, the performance improvements are even more significant because our low-level controller offers foothold adaptation advice directly from real-time visual feedback with lower time latency. Since the heuristic foot placement approach requires a more complex vision system for map construction, we again observe that our hierarchical vision system achieves better timeliness and reliability with minimal hardware systems.

The RL baseline exhibits inferior performance in navigation metrics (SPL, Success) compared with both the proposed method and the heuristic baseline. These results suggest that utilizing successful adaptation labels is a more effective approach than model-free RL methods in obstacle avoidance. Compared to the RL baseline, the proposed method can be smoothly integrated with the high-level navigation policy and the model-based controller. Meanwhile, the RL baseline shows similar performance in both static and dynamic tasks, indicating its ability to handle real-time scenarios effectively.

In the experiments with dynamic targets (shown in Table 3), the proposed hierarchical vision navigation system also achieves the highest success rate compared with the baselines. However, the success rate declines significantly compared with the result with static targets, while the collision rate changes little. This is because the robot has to explore the unknown scenes more and may be unable to find the path to the target. Meanwhile, the Average Collisions change little compared with the experiments with static targets, indicating that the robot maintains a good obstacle avoidance performance with our foothold adaptation method.

#### 5.3.1. Improvements with Hierarchical Vision System

**Figure 7:**
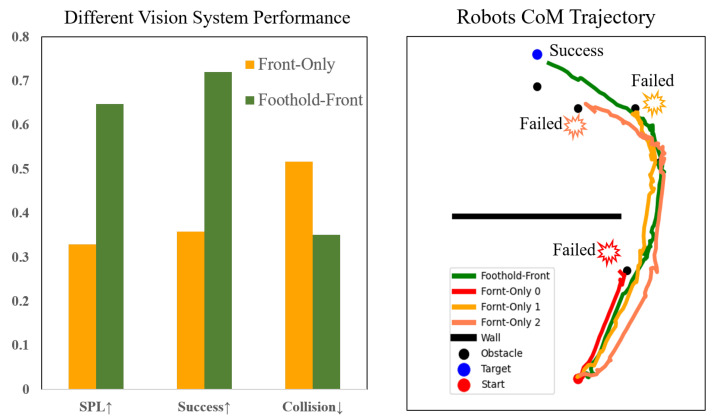
(**left**): The performances of different visual systems. The Foothold-Front system achieves a higher Success Rate and SPL, and lower Average Collisions. (**right**): Center of Mass (COM) trajectories of the compared systems with the same static environment. Due to the lack of foothold observation, the Front-Only system falls at challenging obstacles.

We conduct ablation experiments on the proposed hierarchical vision system. We compare the navigation performance between the proposed system (Foothold-Front) and the system with a single camera (Front-Only). The result is shown in Figure 7. The Foothold-Front system significantly outperforms the Front-Only system. The latter either fails at the challenging obstacles or makes a detour to avoid the obstacles that it could have passed with dynamic foothold adaptation.

#### 5.3.2. Improvements with the Auto-Annotated Supervised Module

This part analyzes the importance of auto-annotated supervised training within the low-level locomotion controller. To eliminate the effects of the high-level navigation policy, we set the experiment in a 5 m straight path where the robot moves forward at a constant velocity of 0.3 m/s. Challenging obstacles are generated in the same way as in the previous experiment. We compare the performance of the foothold adaptation policy trained through auto-annotated supervised under five different data volumes (n-step represents the number of successful steps). As shown in Table 4, the performance improves significantly with the expansion of the auto-labeled data, and the lightweight policy converges in about 1000 steps.

### 5.4. Real-World Experiments

**Figure 8:**
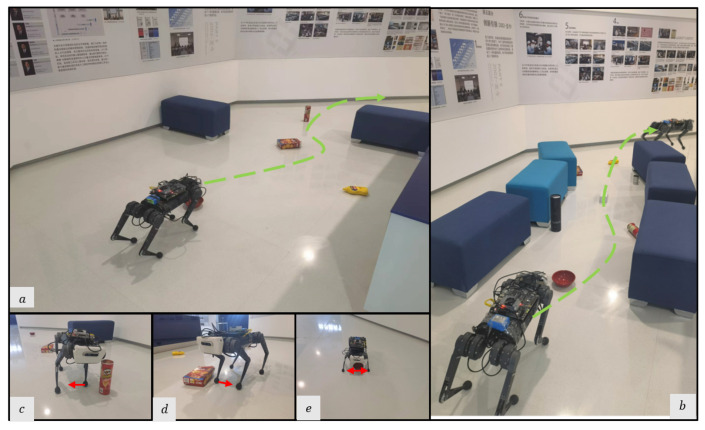
Snapshots of the real robot’s navigation in the cluttered exhibition hall. (**a**,**b**) Optimal path overview in the exhibition hall of different experiments. (**c**–**e**) To side-step the challenging obstacles, the robot selects feasible foot placement locations in different directions from the Original Foothold.

**Figure 9:**
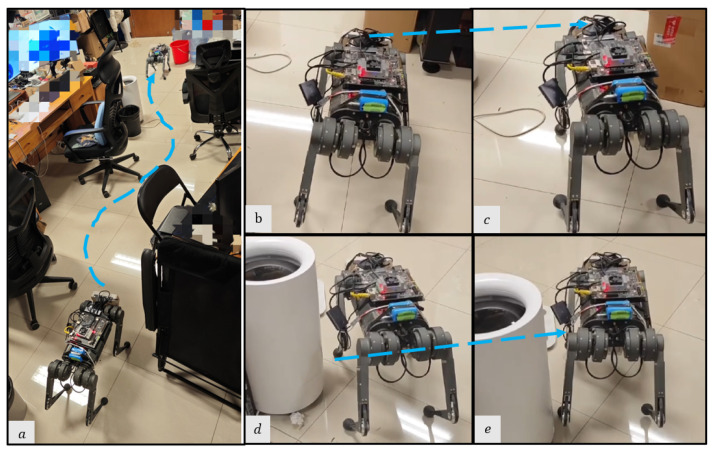
Snapshots of the real robot’s navigation in the static lab environment. (**a**) Optimal path overview in the lab environment; the robot takes a detour through the cluttered narrow path. (**b**,**c**) The Front Camera perceives larger obstacles (the cardboard box) and the high-level policy generates appropriate COM velocity for obstacle avoidance. (**d**,**e**) The Foothold Camera perceives small obstacles (the weight scale) and the foothold adaptation policy generates a feasible foothold for obstacle avoidance.

**Figure 10:**
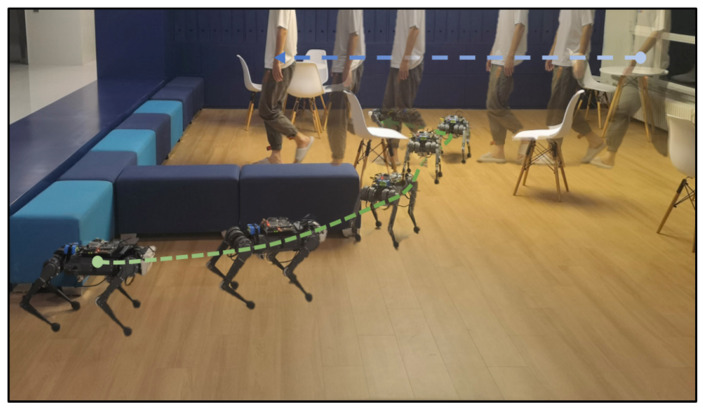
Snapshots of the real robot’s navigation in the parlor with dynamic obstacles; the robot plans an optimal Path overview in the parlor, and a deliberate deviation from the goal direction is introduced to enable the robot to avoid oncoming obstacles.

We deploy the proposed system in a variety of real-world experiments and compare them to the base-nav baseline, which uses the Front Camera only without active foothold adaptation. We conduct experiments in various settings, with each setting being evaluated through 10 trials (5 for each method):

(1) A cluttered exhibition hall (Figure 8) with static obstacles and target points [6 m, 2 m]. To mimic the obstacles in the simulation, we manually construct this scene in the real world. The obstacles include square sofas [0.8 m, 0.4 m, 0.4 m] and YCB objects with maximum size [0.25 m, 0.15 m, 0.1 m].
(2) A static lab environment (Figure 9) with target point [6.5 m, 2 m]. The obstacles are common objects that are naturally placed in indoor scenes, including chairs, desks, the water dispenser, and so on.
(3) A parlor (Figure 10) with target point [5 m, 3 m]. In addition to static obstacles (square sofas, chairs), we incorporate a dynamic obstacle, where a person walks from the side of the robot to its front at an approximate speed of 0.5 m/s. This setting allows us to evaluate the system’s performance in dynamic environments.

#### 5.4.1. Sim-to-Real Transfer

This part provides a clear description of the techniques that we implemented to ensure a successful simulation-to-real transfer.

During simulation, the robot receives task observation $\left(v_{\mathrm{cur}},\theta_{\mathrm{cur}},x_{\mathrm{loc}}\right)$ directly from the simulator, which provides ground-truth information. However, for real-world experiments, the state estimator is used to obtain task observation from the IMU feedback and joint information. The estimator combines raw acceleration, angular velocity, robot orientation, joint position, joint velocity, and an estimated foot contact phase to estimate the robot’s position and velocity. In our work, we use a Kalman Filter-based state estimator [53], which has an average estimation error of 12.2% in our State Estimator tests for a 10 m walk. Similar to the simulation evaluation, the success of navigation is defined by the robot reaching the target point within approximately 1 m. Therefore, we consider this error to be within an acceptable range.

Moreover, we employ domain randomization in both policies. Specifically, we added 1% Gaussian noise to the inputs and outputs involved in the policies, including proprioception states, vision observations, generated COM velocity, and foothold adaptation actions. Furthermore, we pass the COM velocity through the velocity smoother from the ROS (Robot Operating System), which helps to improve the velocity tracking performance of the real robot and prevents sudden motion changes that may cause damage.

#### 5.4.2. Avoiding Static Obstacles

As shown in Table 5, the proposed method achieves a success rate of 0.8 in the cluttered exhibition experiments. When encountering an obstacle in its foothold observation, it tends to adjust the foot location of the nearby leg perpendicular to the direction of movement while preserving the swing trajectories of the other legs. On the other hand, the base-nav method only had a success rate of 0.2, resulting in collisions with all the small obstacles it encountered.

In the static lab environment, the proposed method achieves a success rate of 1.0 and the base-nav method has a success rate of 0.6. The base-nav is more prone to being tripped up and failing when encountering small obstacles such as chair legs and weight scales. The results demonstrate the successful integration of the foothold adaptation policy with the low-level locomotion controller, leading to stable obstacle avoidance in indoor navigation.

#### 5.4.3. Avoiding Dynamic Obstacles

In the parlor with the dynamic obstacle (walking person), the proposed method has a success rate of 0.6 (Table 5) while the base-nav fails for all five trails. We find that the person appearing from the side poses a more challenging obstacle. With base-nav, despite adjusting COM speed upon detecting the person, the robot still becomes tripped by its leg. However, the foothold adaptation policy overcomes such obstacles; the robot is able to react quickly to the upcoming obstacle, and adjust its foothold and COM velocity based on observations from the proposed vision system.

## 6. Conclusions

In this work, we proposed a hierarchical vision navigation system for quadruped robots in cluttered and dynamic scenes. The system is composed of a Front Camera and a Foothold Camera to simultaneously collect visual data from the front and the foothold. The system learns an end-to-end navigation policy at the high level, and enhances the low-level locomotion controller with a foothold adaptation policy through auto-annotated supervised learning. The proposed system opens up future avenues for designing a vision acquisition system to improve the performance of legged robot navigation in unstructured environments.

In future work, we will extend our robot system to tackle challenging environments such as rough terrain. Additionally, we will explore the integration of advanced sensor technologies and perception algorithms to improve the system’s ability to handle complex and dynamic environments, ensuring reliable long-distance navigation, both indoors and outdoors.

## Figures and Tables

**Table 1:** Implementation Hyperparameters.

Policy	Parameter	Value
High-Level Navigation	Discount factor γ	0.99
	Learning rate	1 × 10^−4^
	Batch size	4096
	GAE λ	0.9
	max steps per iter	500
Foothold Adaptation	Pretrained	True
	Greedy factor ϵ	0.75
	Learning Rate	1 × 10^−4^
	Batch Size	32
Motion Controller	Control Frequency	500
	Sim [kp, kd]	[3, 1]
	Real [kp, kd]	[10, 0.2]

**Table 2:** Hierarchical Vision Navigation System Evaluation. Bold values indicate best results.

Scene	Policy ^1^	SPL	Success	Collisions
Static	Base-Nav [46]	0.27	0.29	0.63
	Heuristic [36]	0.64	0.71	0.39
	RL [41]	0.41	0.47	0.44
	Ours	**0.65**	**0.72**	**0.35**
Dynamic	Base-Nav [46]	0.26	0.28	0.63
	Heuristic [36]	0.45	0.50	0.51
	RL [41]	0.45	0.49	0.48
	Ours	**0.57**	**0.65**	**0.39**

^1^ We implement the baseline methods in the same experimental environments for comparison.

**Table 3:** Dynamic Targets Evaluation. Bold values indicate best results.

Obstacles	Policy	Success	Crashes	Collisions
Static	Base-Nav [46]	0.25	0.57	0.63
	Heuristic [36]	0.59	0.21	0.41
	RL [41]	0.42	0.49	0.46
	Ours	**0.62**	**0.20**	**0.38**
Dynamic	Base-Nav [46]	0.29	0.56	0.6
	Heuristic [36]	0.45	0.39	0.46
	RL [41]	0.42	0.51	0.50
	Ours	**0.54**	**0.34**	**0.41**

**Table 4:** Performance of different steps.

n-Step	Static		Dynamic	
	Success	Collisions	Success	Collision
100	0.19	0.66	0.19	0.59
300	0.19	0.62	0.26	0.55
500	0.50	0.41	0.56	0.29
900	0.79	0.26	0.80	0.18
1500	0.75	0.27	0.79	0.21

**Table 5:** Success Rate for Real-World Experiments. Bold values indicate best results.

Policy	Exhibition Hall (Static)	Lab Environment (Static)	Parlor (Dynamic)
Base-Nav	0.2	0.6	0
Ours	**0.8**	**1**	**0.6**

## Data Availability

The data presented in this study are available upon request from the corresponding author.

## Supplementary Materials

The following supporting information can be downloaded at: https://www.mdpi.com/article/10.3390/s23115194/s1, Video S1: Supplementary Videos.

## References

[1] Roscia F., Cumerlotti A., Del Prete A., Semini C., Focchi M. (2023). Orientation Control System: Enhancing Aerial Maneuvers for Quadruped Robots. Sensors.

[2] Semini C. (2010). HyQ-Design and Development of a Hydraulically Actuated Quadruped Robot. Ph.D. Thesis.

[3] Bonin-Font F., Ortiz A., Oliver G. (2008). Visual navigation for mobile robots: A survey. J. Intell. Robot. Syst..

[4] Durrant-Whyte H., Bailey T. (2006). Simultaneous localization and mapping: Part I. IEEE Robot. Autom. Mag..

[5] Fuentes-Pacheco J., Ruiz-Ascencio J., Rendón-Mancha J.M. (2015). Visual simultaneous localization and mapping: A survey. Artif. Intell. Rev..

[6] Thrun S. (2002). Probabilistic robotics. Commun. ACM.

[7] Bansal S., Tolani V., Gupta S., Malik J., Tomlin C. Combining optimal control and learning for visual navigation in novel environments. Proceedings of the Conference on Robot Learning, PMLR.

[8] Truong J., Yarats D., Li T., Meier F., Chernova S., Batra D., Rai A. Learning navigation skills for legged robots with learned robot embeddings. Proceedings of the 2021 IEEE/RSJ International Conference on Intelligent Robots and Systems (IROS).

[9] Ravankar A., Ravankar A.A., Kobayashi Y., Hoshino Y., Peng C.C. (2018). Path smoothing techniques in robot navigation: State-of-the-art, current and future challenges. Sensors.

[10] Fahmi S., Mastalli C., Focchi M., Semini C. (2019). Passive Whole-Body Control for Quadruped Robots: Experimental Validation Over Challenging Terrain. IEEE Robot. Autom. Lett..

[11] Ding Y., Pandala A., Park H.W. Real-time Model Predictive Control for Versatile Dynamic Motions in Quadrupedal Robots. Proceedings of the 2019 International Conference on Robotics and Automation (ICRA).

[12] Neunert M., Stäuble M., Giftthaler M., Bellicoso C.D., Carius J., Gehring C., Hutter M., Buchli J. (2018). Whole-body nonlinear model predictive control through contacts for quadrupeds. IEEE Robot. Autom. Lett..

[13] Miki T., Lee J., Hwangbo J., Wellhausen L., Koltun V., Hutter M. (2022). Learning robust perceptive locomotion for quadrupedal robots in the wild. Sci. Robot..

[14] Gangapurwala S., Geisert M., Orsolino R., Fallon M., Havoutis I. (2022). Rloc: Terrain-aware legged locomotion using reinforcement learning and optimal control. IEEE Trans. Robot..

[15] Wellhausen L., Dosovitskiy A., Ranftl R., Walas K., Cadena C., Hutter M. (2019). Where should i walk? predicting terrain properties from images via self-supervised learning. IEEE Robot. Autom. Lett..

[16] Fankhauser P., Bjelonic M., Bellicoso C.D., Miki T., Hutter M. Robust rough-terrain locomotion with a quadrupedal robot. Proceedings of the 2018 IEEE International Conference on Robotics and Automation (ICRA).

[17] Moreno F.A., Monroy J., Ruiz-Sarmiento J.R., Galindo C., Gonzalez-Jimenez J. (2019). Automatic waypoint generation to improve robot navigation through narrow spaces. Sensors.

[18] Zhang Y., Zhou Y., Li H., Hao H., Chen W., Zhan W. (2022). The Navigation System of a Logistics Inspection Robot Based on Multi-Sensor Fusion in a Complex Storage Environment. Sensors.

[19] Li Y., Dai S., Shi Y., Zhao L., Ding M. (2019). Navigation simulation of a Mecanum wheel mobile robot based on an improved A* algorithm in Unity3D. Sensors.

[20] Ali M.A., Mailah M. (2019). Path planning and control of mobile robot in road environments using sensor fusion and active force control. IEEE Trans. Veh. Technol..

[21] Wang S., Zhang H., Wang G. (2022). OMC-SLIO: Online Multiple Calibrations Spinning LiDAR Inertial Odometry. Sensors.

[22] Dudzik T., Chignoli M., Bledt G., Lim B., Miller A., Kim D., Kim S. Robust autonomous navigation of a small-scale quadruped robot in real-world environments. Proceedings of the 2020 IEEE/RSJ International Conference on Intelligent Robots and Systems (IROS).

[23] Engel J., Schöps T., Cremers D. (2014). LSD-SLAM: Large-scale direct monocular SLAM. Proceedings of the European Conference on Computer Vision.

[24] Mur-Artal R., Montiel J.M.M., Tardos J.D. (2015). ORB-SLAM: A versatile and accurate monocular SLAM system. IEEE Trans. Robot..

[25] Engel J., Koltun V., Cremers D. (2017). Direct sparse odometry. IEEE Trans. Pattern Anal. Mach. Intell..

[26] Díaz-Vilariño L., Khoshelham K., Martínez-Sánchez J., Arias P. (2015). 3D modeling of building indoor spaces and closed doors from imagery and point clouds. Sensors.

[27] Pfeiffer M., Schaeuble M., Nieto J., Siegwart R., Cadena C. From perception to decision: A data-driven approach to end-to-end motion planning for autonomous ground robots. Proceedings of the 2017 IEEE International Conference on Robotics and Automation (ICRA).

[28] Wijmans E., Kadian A., Morcos A., Lee S., Essa I., Parikh D., Savva M., Batra D. (2019). Dd-ppo: Learning near-perfect pointgoal navigators from 2.5 billion frames. arXiv.

[29] Cetin E., Barrado C., Munoz G., Macias M., Pastor E. Drone navigation and avoidance of obstacles through deep reinforcement learning. Proceedings of the 2019 IEEE/AIAA 38th Digital Avionics Systems Conference (DASC).

[30] Pandey A., Pandey S., Parhi D. (2017). Mobile robot navigation and obstacle avoidance techniques: A review. Int. Rob. Auto J..

[31] Zhao X., Agrawal H., Batra D., Schwing A.G. The surprising effectiveness of visual odometry techniques for embodied pointgoal navigation. Proceedings of the IEEE/CVF International Conference on Computer Vision.

[32] Li T., Calandra R., Pathak D., Tian Y., Meier F., Rai A. (2021). Planning in learned latent action spaces for generalizable legged locomotion. IEEE Robot. Autom. Lett..

[33] Hoeller D., Wellhausen L., Farshidian F., Hutter M. (2021). Learning a state representation and navigation in cluttered and dynamic environments. IEEE Robot. Autom. Lett..

[34] Fu Z., Kumar A., Agarwal A., Qi H., Malik J., Pathak D. Coupling vision and proprioception for navigation of legged robots. Proceedings of the IEEE/CVF Conference on Computer Vision and Pattern Recognition.

[35] Kim D., Di Carlo J., Katz B., Bledt G., Kim S. (2019). Highly dynamic quadruped locomotion via whole-body impulse control and model predictive control. arXiv.

[36] Kim D., Carballo D., Di Carlo J., Katz B., Bledt G., Lim B., Kim S. Vision Aided Dynamic Exploration of Unstructured Terrain with a Small-Scale Quadruped Robot. Proceedings of the 2020 IEEE International Conference on Robotics and Automation (ICRA).

[37] Agrawal A., Chen S., Rai A., Sreenath K. Vision-aided dynamic quadrupedal locomotion on discrete terrain using motion libraries. Proceedings of the 2022 International Conference on Robotics and Automation (ICRA).

[38] Hwangbo J., Lee J., Dosovitskiy A., Bellicoso D., Tsounis V., Koltun V., Hutter M. (2019). Learning agile and dynamic motor skills for legged robots. Sci. Robot..

[39] Yang C., Yuan K., Zhu Q., Yu W., Li Z. (2020). Multi-expert learning of adaptive legged locomotion. Sci. Robot..

[40] Ji G., Mun J., Kim H., Hwangbo J. (2022). Concurrent Training of a Control Policy and a State Estimator for Dynamic and Robust Legged Locomotion. IEEE Robot. Autom. Lett..

[41] Yu W., Jain D., Escontrela A., Iscen A., Xu P., Coumans E., Ha S., Tan J., Zhang T. Visual-locomotion: Learning to walk on complex terrains with vision. Proceedings of the 5th Annual Conference on Robot Learning.

[42] Kumar A., Fu Z., Pathak D., Malik J. (2021). Rma: Rapid motor adaptation for legged robots. arXiv.

[43] Lee J., Hwangbo J., Wellhausen L., Koltun V., Hutter M. (2020). Learning quadrupedal locomotion over challenging terrain. Sci. Robot..

[44] Peng X.B., Coumans E., Zhang T., Lee T.W., Tan J., Levine S. (2020). Learning agile robotic locomotion skills by imitating animals. arXiv.

[45] Anderson P., Chang A., Chaplot D.S., Dosovitskiy A., Gupta S., Koltun V., Kosecka J., Malik J., Mottaghi R., Savva M. (2018). On evaluation of embodied navigation agents. arXiv.

[46] Xia F., Shen W.B., Li C., Kasimbeg P., Tchapmi M.E., Toshev A., Martín-Martín R., Savarese S. (2020). Interactive gibson benchmark: A benchmark for interactive navigation in cluttered environments. IEEE Robot. Autom. Lett..

[47] Schulman J., Wolski F., Dhariwal P., Radford A., Klimov O. (2017). Proximal policy optimization algorithms. arXiv.

[48] Liang E., Liaw R., Nishihara R., Moritz P., Fox R., Goldberg K., Gonzalez J., Jordan M., Stoica I. RLlib: Abstractions for distributed reinforcement learning. Proceedings of the International Conference on Machine Learning.

[49] Villarreal O., Barasuol V., Camurri M., Focchi M., Franceschi L., Pontil M., Caldwell D., Semini C. (2019). Fast and continuous foothold adaptation for dynamic locomotion through convolutional neural networks. IEEE Robot. Autom. Lett..

[50] Coumans E., Bai Y. (2016). Pybullet, a Python Module for Physics Simulation for Games, Robotics and Machine Learning. http://pybullet.org/.

[51] Xiang Y., Schmidt T., Narayanan V., Fox D. (2017). PoseCNN: A Convolutional Neural Network for 6D Object Pose Estimation in Cluttered Scenes. arXiv.

[52] He K., Zhang X., Ren S., Sun J. Deep residual learning for image recognition. Proceedings of the IEEE Conference on Computer Vision and Pattern Recognition.

[53] Bloesch M., Hutter M., Hoepflinger M.A., Leutenegger S., Gehring C., Remy C.D., Siegwart R. (2013). State estimation for legged robots-consistent fusion of leg kinematics and IMU. Robotics.

